# Potential identification of vitamin B6 responsiveness in autism spectrum disorder utilizing phenotype variables and machine learning methods

**DOI:** 10.1038/s41598-018-33110-w

**Published:** 2018-10-04

**Authors:** Taku Obara, Mami Ishikuro, Gen Tamiya, Masao Ueki, Chizuru Yamanaka, Satoshi Mizuno, Masahiro Kikuya, Hirohito Metoki, Hiroko Matsubara, Masato Nagai, Tomoko Kobayashi, Machiko Kamiyama, Mikako Watanabe, Kazuhiko Kakuta, Minami Ouchi, Aki Kurihara, Naru Fukuchi, Akihiro Yasuhara, Masumi Inagaki, Makiko Kaga, Shigeo Kure, Shinichi Kuriyama

**Affiliations:** 1 Tohoku Medical Megabank Organization (ToMMo), Tohoku University, Sendai, Miyagi Japan; 2 Department of Molecular Epidemiology, Graduate School of Medicine, Tohoku University, Sendai, Miyagi Japan; 3 Department of Pharmaceutical Sciences, Tohoku University Hospital, Sendai, Miyagi Japan; 4 Statistical Genetics Team, RIKEN Center for Advanced Intelligence Project, Chuo-ku, Tokyo, Japan; 5 Department of Hygiene and Public Health, School of Medicine, Teikyo University, Tokyo, Japan; 6 Division of Public Health, Hygiene and Epidemiology, Tohoku Medical and Pharmaceutical University, Sendai, Miyagi Japan; 7 Department of Pediatrics, Graduate School of Medicine, Tohoku University, Sendai, Miyagi Japan; 8 Department of Education, Art and Science, Yamagata University, Yamagata, Yamagata, Japan; 9 Department of Pediatrics, Saka General Hospital, Shiogama, Miyagi Japan; 10 Kakuta Child & Allergy Clinic, Tagajo, Miyagi Japan; 11 Department of Pediatrics, NTT Medical Center Tokyo, Shinagawa-ku, Tokyo, Japan; 12 Bunkyo Education Center, Bunkyo-ku, Tokyo, Japan; 13 Fujimoto Shinjuku Hospital, Shinjuku-ku, Tokyo, Japan; 14 Department of Psychiatry, Miyagi Psychiatric Center, Natori, Miyagi Japan; 15 Miyagi Disaster Mental Health Care Center, Sendai, Miyagi Japan; 16 Yasuhara Children’s Clinic, Neyagawa, Osaka, Japan; 17 Department of Developmental Disorders, National Institute of Mental Health, National Center of Neurology and Psychiatry, Kodaira, Tokyo, Japan; 18 Tokyo Metropolitan Tobu Medical Center for Children with Developmental Disabilities, Koto-ku, Tokyo, Japan; 19 Department of Disaster Public Health, International Research Institute of Disaster Science, Tohoku University, Sendai, Miyagi Japan

## Abstract

We investigated whether machine learning methods could potentially identify a subgroup of persons with autism spectrum disorder (ASD) who show vitamin B6 responsiveness by selected phenotype variables. We analyzed the existing data from our intervention study with 17 persons. First, we focused on signs and biomarkers that have been identified as candidates for vitamin B6 responsiveness indicators. Second, we conducted hypothesis testing among these selected variables and their combinations. Finally, we further investigated the results by conducting cluster analyses with two different algorithms, affinity propagation and k-medoids. Statistically significant variables for vitamin B6 responsiveness, including combination of hypersensitivity to sound and clumsiness, and plasma glutamine level, were included. As an a priori variable, the Pervasive Developmental Disorders Autism Society Japan Rating Scale (PARS) scores was also included. The affinity propagation analysis showed good classification of three potential vitamin B6-responsive persons with ASD. The k-medoids analysis also showed good classification. To our knowledge, this is the first study to attempt to identify subgroup of persons with ASD who show specific treatment responsiveness using selected phenotype variables. We applied machine learning methods to further investigate these variables’ ability to identify this subgroup of ASD, even when only a small sample size was available.

## Introduction

Autism spectrum disorder (ASD) is a disorder characterized by difficulties in social interaction and communication, and repetitive behaviours^1^. In recent years, ASD has been investigated using advanced technologies such as machine learning to improve diagnosis and prognosis prediction^2,3^. Machine learning employs artificial intelligence techniques to discover useful masked patterns.

The effects of vitamin B6 in ASD are controversial. Previous attempts to evaluate the efficacy of vitamin B6 in treating persons with ASD have failed to produce consistent findings^4–8^. ASD is highly heterogeneous^9^, and identifying a subgroup of persons who may show responsiveness to vitamin B6, if any, is important.

We previously identified similarities between ASD and pyridoxine-dependent epilepsy (PDE)^10^, which are generally considered quite different. Pyridoxine is one form of vitamin B6. Many of the signs of PDE parallel those of ASD. These signs include epilepsy, autistic tendency, hypersensitivity to sound, expressive verbal disorders, and clumsiness^1,11,12^. Therefore, we hypothesized that some persons with ASD share, even in part, some etiologies with persons with PDE and that this subgroup of persons with ASD also responds to vitamin B6 intervention. We previously conducted a preliminary randomized controlled trial to evaluate the efficacy of a high-dose vitamin B6 treatment in persons with ASD who exhibited hypersensitivity to sound, expressive verbal disorders, and clumsiness, and we found that the intervention improved verbal IQ^10,13^. Our preliminary results indicate that hypersensitivity to sound, expressive verbal disorders, and clumsiness might predict responsiveness to vitamin B6.

In addition to signs, biomarkers might also be candidates contributing to the classification of ASD. Several previous studies have consistently suggested the presence of increased plasma amino acids of glutamate and decreased glutamine among persons with some ASD^14–16^. These findings suggest that plasma glutamate and glutamine levels might contribute to the classification of ASD in addition to signs.

In the present study, we explored whether the signs and biomarkers described above can be used to classify vitamin B6-responsive persons with ASD from nonresponsive ASD persons. We apply machine learning methods of affinity propagation (AP)^17^ and k-medoids^18^ to further investigate these variables’ ability to identify a subgroup of persons with ASD.

## Results

### Characteristics of participants

The participants’ pre-intervention characteristics are shown in Table 1. The 17 participants included 13 boys and 4 girls. The mean age was 8.8 years (standard deviation, 4.1 years). Sixteen of 17 participants showed expressive verbal disorders.

**Table 1:** Characteristics and selected signs of participants before intervention.

Boys/Girls (No.)	13/4
Age (years) (SD)	8.8 (4.1), range; 5–19
Body weight (kg) (SD)	32.8 (15.6), range; 15.6–62.0
Body height (cm) (SD)	130.6 (21.8), range; 98.2–170.0
Signs (No.)	
Hypersensitivity to sound	8/17
Expressive verbal disorders	16/17
Clumsiness	10/17

No., number. SD, standard deviation.

### Hypothesis testing

Three participants were classified as vitamin B6 possible responders, whereas 14 were classified as less responders. All three possible responders showed coexisting hypersensitivity to sound and clumsiness, compared with only two of the 14 less responders, representing a statistically significant difference (P = 0.01) (Table 2).

**Table 2:** Number of participants according to signs and potential vitamin B6 responsiveness.

Signs	Potential vitamin B6 responsiveness^a^		P-value^b^
	“possible responders”	“less responders”	
Hypersensitivity to sound			
Yes	3	5	0.08
No	0	9	
Clumsiness			
Yes	3	7	0.18
No	0	7	
Hypersensitivity to sound and clumsiness			
Yes	3	2	0.01
No	0	12	

^a^According to the Clinical Global Impression-Improvement scale.

^b^Fisher’s exact test.

A comparison of plasma amino acids levels between the possible responders and less responders showed large differences in glutamine levels (reference range: 420–700 nmol/mL). The mean glutamine levels measured before the vitamin B6 treatment were 400.5 nmol/mL (standard error (SE), 11.9 nmol/mL) in the possible responders and 481.4 nmol/mL (SE, 10.4 nmol/mL) in the less responders; this difference was significant (P = 0.004) (Table 3).

**Table 3:** Differences in selected plasma amino acids levels according to potential vitamin B6 responsiveness.

Variables	Potential vitamin B6 responsiveness^a^	Plasma levels (SE)	P-value^b^
Glutamate (nmol/mL)	“possible responders”	21.5 (3.0)	0.86
	“less responders”	20.5 (2.4)	
Glutamine (nmol/mL)	“possible responders”	400.5 (11.9)	0.004
	“less responders”	481.4 (10.4)	

SE, standard error.

^a^According to the Clinical Global Impression-Improvement scale.

^b^Student *t*-test.

### Cluster analysis

**Figure 1:**
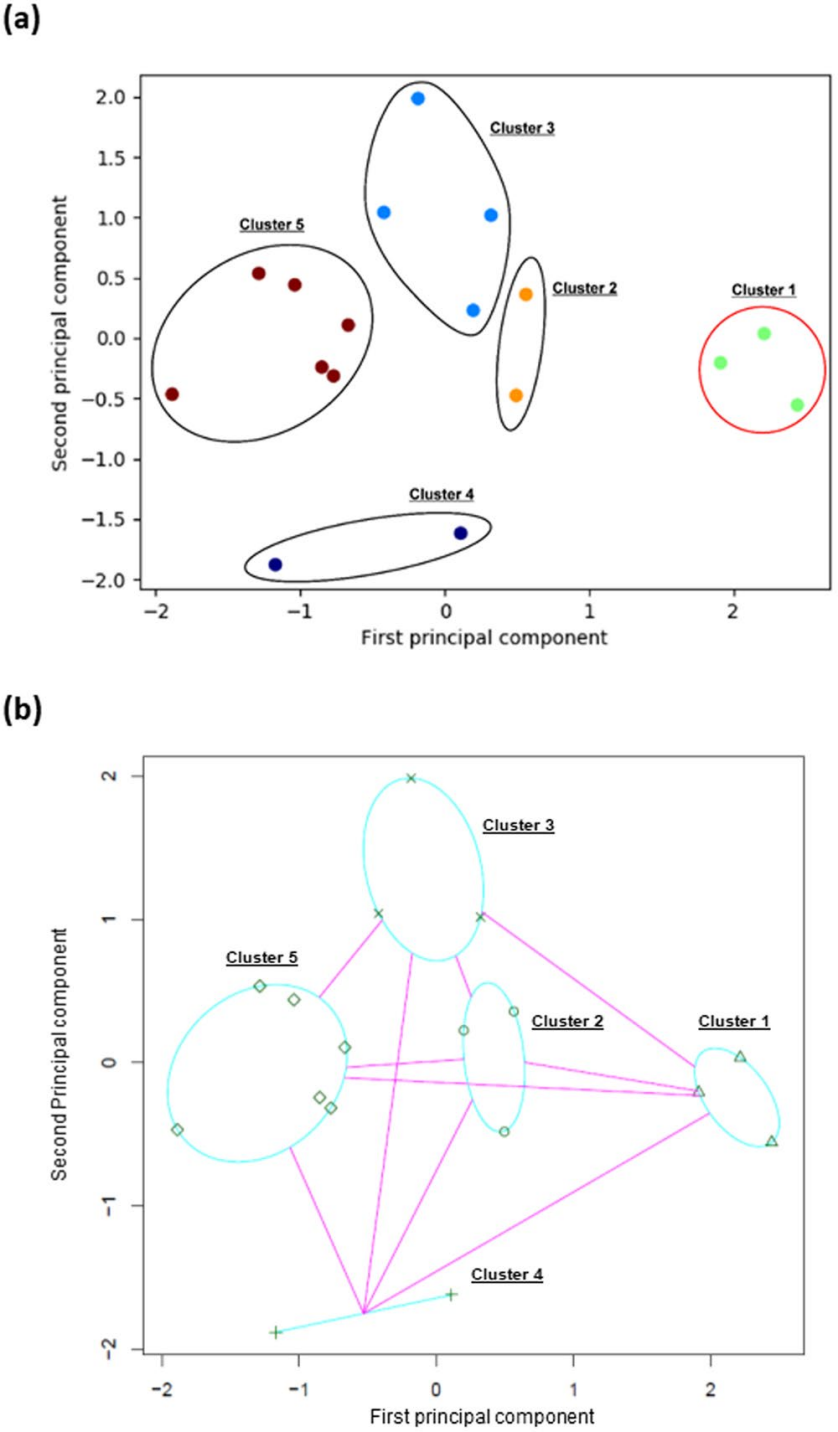
A graphical representation of the affinity propagation (**a**) and k-medoids (**b**) clustering results using principal component analysis. The affinity propagation (AP) analysis showed good classifying of potential vitamin B6-responsive persons with ASD (cluster 1). All the participants were relatively well classified into five groups, and clusters 2 to 5 consisted of persons who exhibited a low response to vitamin B6. A graphical representation of the AP clustering results using principal component analysis (PCA) is presented in **1a**. The k-medoids analysis also showed good classification . The selected number of clusters by k-medoids was also five and the result was identical to that by the AP except for one participants who was classified in Cluster 2 by the AP was classified in Cluster 3 by the k-medoids method. A graphical representation of the k-medoids clustering results using PCA is presented in **1b**.

Using the presence of hypersensitivity to sound concomitant with the presence of clumsiness, plasma glutamine levels, and the Pervasive Developmental Disorders Autism Society Japan Rating Scale (PARS)^19^ scores, the AP analysis showed good classification of potential vitamin B6-responsive persons with ASD (cluster 1). All the participants were relatively well classified into five groups, and clusters 2 to 5 consisted of persons who exhibited a low response to vitamin B6. A graphical representation of the AP clustering results using principal component analysis (PCA)^20^ is presented in Fig. 1a. The mean glutamine levels before the vitamin B6 treatment were 495.6 nmol/mL in cluster 2, 476.3 nmol/mL in cluster 3, 417.8 nmol/mL in cluster 4, and 501.2 nmol/mL in cluster 5, respectively.

The k-medoids analysis also showed good classification. The selected number of clusters by k-medoids was also five and the result was identical to that by AP except for one participants who was classified into cluster 2 by AP and into cluster 3 by the k-medoids method. A graphical representation of the k-medoids clustering results using PCA is presented in Fig. 1b.

## Discussion

Using the machine learning clustering algorithms of AP and k-medoids, we successfully classified an ASD subgroup that responded to vitamin B6 from others by selected phenotype variables. The additional k-medoids analysis reflected the robustness of our clustering result because the k-medoids method is completely different from AP. Our data indicate that common variables used in previous studies may be able to identify subgroup that exhibit responsiveness to specific treatment.

Our sample size is relatively small. Because we selected candidate phenotype variables according to evidence from previous studies, they are free from the multiplicity of hypothesis tests. Furthermore, contrary to hypothesis testing, cluster analysis does not necessarily require a large sample size because it separates data according to structures. In cluster analyses, distance between clusters is more important for stable clustering than sample sizes.

Variable selection is critical issues in clustering analysis. In this study, we focused on signs and biomarkers that have been identified in previous studies as candidates and conducted hypothesis testing among these selected variables and their combinations to further select relevant variables and to reduce the dimension. Recent medical studies have frequently used the method of reducing dimension and subsequent clustering. Based on the clinical and/or statistical approach, researchers identify subsets or patterns of variables to address their study aims^21,22^. We believe that our protocol is one appropriate strategy to identify subgroup of diseases including ASD.

AP is an unsupervised clustering algorithm that identifies clusters of similar points using a set of points and a set of similarity values between the points and provides a representative example, called an exemplar, for each cluster^17^. We identified five clusters from 17 persons with ASD using the AP analysis. Of the five clusters, cluster 1 consisted of all persons who responded to vitamin B6. Although we could not identify meaningful characteristics of the other four clusters (clusters 2 to 5), the AP analysis suggested that persons with ASD may be highly heterogeneous, as previously reported^9^.

Cluster analysis classifies data into groups according to their structure. However, cluster analysis itself does not predict a sample as a specific cluster. Although our cluster analyses could potentially identify a subgroup of ASD, their predictive value requires further analyses utilizing different algorithms, such as support vector machine^23,24^, and evaluation of the accuracy of these results. Based on our data, further studies to determine the predictive value are warranted, presumably with a larger sample size and validation data sets.

Why low levels of plasma glutamine levels predict vitamin B6 responsiveness is unclear. Glutamine plays a central role in nitrogen metabolism in many cell systems and in the central nervous system, and glutamine synthesis has a neuroprotective function because it removes ammonia and glutamate^25^. Vitamin B6 also plays a predominant role as a coenzyme for these substances^26^. Therefore, we speculate that consuming a high dose of vitamin B6 may improve the activity of the metabolic pathways described above.

The prevalence of pyridoxine responsiveness is unknown. Although three of the 17 participants exhibited a possible response to vitamin B6 in our intervention study, the study population was not randomly recruited from among persons with ASD, rather, we restrictively recruited the participants with potential responsiveness. Therefore, the proportion of vitamin B6 responders in the general population with ASD may be relatively small. Unfortunately, in daily practice, clinically used ASD assessment tools generally do not include hypersensitivity to sound or clumsiness, and clinical tests do not typically include serum or plasma glutamine levels. Therefore, more data may be necessary to assess vitamin B6 responsiveness and estimate its prevalence.

We might be able to identify the genetic factors responsible for vitamin B6-responsive ASD. Although the high heritability of ASD is supported by high concordance rates (from 36% to 95%) in monozygotic twins and a higher recurrence risk of 11% and 19% with single-sibling involvement^27–29^, no susceptibility genes have been specified in ASD^30^. Based on the results of the present study, the clustering of some signs and biomarkers might be informative and provide the best model for identifying etiologically similar and medically treatable predictive cases of ASD.

The possibility that a low proportion of vitamin B6 responders with ASD and the small sample size were limitations of the present study. Therefore, the present findings should be interpreted with caution, and external validity should be confirmed by further studies.

To our knowledge, this is the first study to attempt to identify subgroup of persons with ASD who show vitamin B6 responsiveness using selected phenotype variables. We applied the machine learning methods of AP and k-medoids to further investigate these variables’ ability to identify a subgroup of ASD, even when only a small sample size was available.

## Methods

### Study design

We analyzed the existing data from our intervention study as follows: 1) we focused on signs and biomarkers that have been identified in previous studies^10,13–16^ as candidates for vitamin B6 responsiveness indicators; 2) we conducted hypothesis testing among these selected variables and their combinations to further select variables and to reduce the dimension; and 3) we conducted cluster analysis to further investigate the variables’ predictive ability using two different algorithms.

### Data characteristics

#### Intervention study

We conducted a single-arm intervention from October 24, 2007 to September 2, 2009. Seventeen persons with ASD were recruited from seven medical institutions in Japan. Medical doctors invited potential vitamin B6 responders who showed hypersensitivity to sound, expressive verbal disorders, and/or clumsiness. The inclusion criteria for participation in this intervention study were as follows: (1) a diagnosis of ASD (code F84) determined by a doctor according to the International Classification of Diseases, 10th Revision (ICD-10)^31^; (2) 5 to 20 years of age; (3) no history of epilepsy; (4) no diagnosis of homocystinuria or fragile X syndrome; and (5) not currently receiving vitamin B6 treatments.

We selected the above age range to attempt to avoid an unstable phenotype for younger children and to exclude the effects of deuteropathy of more elderly persons.

#### Vitamin B6 responsiveness

Participants received 5 mg vitamin B6/kg body weight per day for two weeks followed by 10 mg vitamin B6/kg body weight per day for two weeks, resulting in a total treatment period of four weeks. Using the Clinical Global Impression-Improvement (CGI-I) scale^32^, medical doctors evaluated responsiveness to vitamin B6 as “very much improved,” “much improved,” “minimally improved,” or “no change.” The CGI Scale is a measure of global clinical change with strong validity that has been widely used as an outcome measure in clinical trials of central nervous system disorders^33^. We divided participants into two groups: “possible responders,” who were “very much improved” or “much improved”, and “less responders,” who were “minimally improved” or showed “no change”.

#### Variable measurements

Before the intervention, participants were assessed for signs including hypersensitivity to sound, expressive verbal disorders, and clumsiness, and plasma amino acids including glutamate and glutamine. A person with ASD was considered to show a sign, such as hypersensitivity to sound, when both his/her medical doctor and guardian independently assessed the sign as present. The PARS was used to evaluate autistic traits^19^.

#### Ethical issues

We conducted this intervention study in accordance with the guidelines of the Declaration of Helsinki^34^ and all other applicable guidelines. The protocol was reviewed and approved by the institutional review board of Tohoku University Graduate School of Medicine. The board also reviewed and approved the study protocol for Miyagi Psychiatric Center, Kakuta Child & Allergy Clinic, NTT Medical Center Tokyo, Fujimoto Shinjuku Hospital, Yasuhara Children’s Clinic, and National Center of Neurology and Psychiatry. For participants less than 16 years of age, we obtained informed assent from the individuals and informed consent from their guardians. For participants aged 16 to 19 years, we obtained informed consent from both the individuals and their guardians.

### Hypothesis testing

Regarding the signs’ variables, we excluded expressive verbal disorders in the present analyses because they were observed in 16 of 17 participants. Signs and their combination of hypersensitivity to sound and clumsiness, and also biomarkers of plasma glutamate level, glutamine level were compared between vitamin B6 possible responders and less responders. Signs of the participants were compared using Fisher’s exact test. Student’s *t*-test was used to investigate the significance of the differences in the biomarkers.

The above statistical analyses were performed using SAS version 9.4 (SAS Inc., Cary, NC, USA). We used approximate variance formulas to calculate 95% confidence intervals (CIs). A *P* value < 0.05 was accepted as a statistically significant value. All *P* values were two-tailed.

### Cluster analysis

In the cluster analysis, statistically significant variables for vitamin B6 responsiveness, including hypersensitivity to sound combined with clumsiness, and plasma glutamine level, were included in the preprocessed dataset. As an a priori variable, the PARS score was also included. A relatively recently developed algorithm, AP^17^, was first applied to the preprocessed dataset to divide the participants into vitamin B6 possible-responsive and less-responsive groups. AP is an unsupervised (i.e. without using the information of possible responders and less responders) clustering analysis method using a message-passing-based algorithm. In the present analysis, AP was performed without diagonal components using a dumping factor of 0.9. These analyses were performed with the scikit-learn toolkit in Python 2.6^35,36^.

To examine the robustness of the clustering by AP, we additionally conducted a completely different clustering analysis using k-medoids^18^ implemented in the “fpc” package for R version 3.3.2^37^. It is a fully automated method similar to AP in which the optimal number of clusters is automatically determined by the average silhouette width.

To create a graphical representation of the clustering results, we adopted a PCA^20^.

## Data Availability

The data used in this study will be shared upon request.
